# Dry-Season Soil and Co-Cultivated Host Plants Enhanced Propagation of Arbuscular Mycorrhizal Fungal Spores from Sand Dune Vegetation in Trap Culture

**DOI:** 10.3390/jof8101061

**Published:** 2022-10-10

**Authors:** Ugyen Wangmo Tenzin, Nuttapol Noirungsee, Phanthipha Runsaeng, Prakrit Noppradit, Lompong Klinnawee

**Affiliations:** 1 Division of Biological Science, Faculty of Science, Prince of Songkla University, Songkhla 90110, Thailand; 2 Plant Cell and Physiology for Sustainable Agriculture Research Unit, Faculty of Science, Prince of Songkla University, Songkhla 90110, Thailand; 3 Department of Biology, Faculty of Science, Chiang Mai University, Chiang Mai 50200, Thailand; 4 Research Center in Bioresources for Agriculture, Industry and Medicine, Chiang Mai University, Chiang Mai 50200, Thailand; 5 Division of Health and Applied Sciences, Faculty of Science, Prince of Songkla University, Songkhla 90110, Thailand; 6 Faculty of Environmental Management, Prince of Songkla University, Songkhla 90110, Thailand

**Keywords:** coastal sand dune vegetation, trap culture, AMF spore propagation, co-cultivation of AMF host plants, AMF community composition

## Abstract

The use of arbuscular mycorrhizal fungi (AMF) as biofertilizer in agriculture is a sustainable approach to fertilization. The first step in the production of AMF biofertilizer is inoculation of mycotrophic plants with a composite of soil and native plant roots, containing potentially viable AMF spores from natural habitats, to a trap culture. A single host plant or a consortium of host plants can be used to propagate AMF spores. However, the difference in the comparative efficiency of mono- and co-cultivated host plants used for the production of AMF spores and the maintenance of original AMF community composition has not been well elucidated. Here, we prepared trap culture with nutrient-poor soil from coastal sand dune vegetation collected during the dry season when the AMF spore density and relative abundance of Glomeromycota ITS2 sequences were significantly higher (*p* = <0.05) than in the wet season. The AMF communities in the soil were mainly composed of *Glomus* spp. Maize (*Zea mays* L.) and/or Sorghum (*Sorghum bicolor* (L.). Moench) were grown in trap cultures in the greenhouse. Our results demonstrated that co-cultivation of the host plants increased the production of AMF spores but, compared to mono-cultivation of host plants, did not better sustain the native AMF community compositions in the coastal sand dune soil. We propose that the co-cultivation of host plants in a trap culture broadens AMF-host plant compatibilities and thus sustains the symbiotic association of the natively diverse AMF. Therefore, the results of this study suggest that further research is needed to confirm whether the co-culturing of more than one host plant is as efficient a strategy as using a monoculture of a single host plant.

## 1. Introduction

Arbuscular mycorrhizal fungi (AMF) belonging to the phylum Glomeromycota are obligate root endophytic fungi that form mutualistic associations with almost all terrestrial plants. Under nutrient-limited conditions, the enhanced nutrient acquisition facilitated by networks of AMF hyphae enables host plants to obtain more nutrients, especially phosphorus [1]. Reciprocally, host plants feed AMF with photosynthates, which are primarily sugars and lipids [2]. In addition to enhanced nutrient acquisition, symbiosis with AMF increases host plant tolerance to environmental stresses such as salinity, drought and flooding [3,4,5]. These stresses are three major constraints in agriculture and therefore farmers have long used AMF, in the form of AMF inoculants, as natural biofertilizers [6]. Since the application of AMF biofertilizers can reduce the use of chemical fertilizer, the demand for AMF biofertilizers has gradually increased to sustain the growth in intensive agriculture [7].

The production of AMF inoculants is initiated by the trap culture method [8]. A trap culture is prepared in a pot containing AMF host plants grown in a composite rhizo-spheric soil and/or native plant roots potentially harboring AMF from the habitat of interest. The trap culture is normally maintained in a greenhouse for three months until the propagated batch of AMF spores is collected [8]. The efficiency of trap culture depends on intricate interactions between plant genotype, environment, AMF and agro-ecosystem management [9]. The selection of appropriate AMF host plants is a crucial step that determines the success of AMF spore propagation and a wide variety of AMF host plants have been used in trap cultures. However, the effective use of a host plant species in one trap culture might not guarantee the success of the same species in another trap culture. The difficulty of trap culture has been demonstrated by the inconsistent results observed across different studies, with regard to AMF spore production at the end of the trap culture [10,11]. Due to the particularly specific interactions between AMF species and their environments, the establishment of universally compatible trap plant host species for all AMF species is unlikely. Nevertheless, fast-growing C4 plants, including maize and sorghum, are common host plants used in trap culture due to their high photosynthetic efficiency and resilience to environmental disturbances [8,12].

To improve the efficiency of trap culture, a modification of the host plant management was introduced by the use of a host plant consortium consisting of *Plantago lanceolata*, *Trifolium pratense* and *Lolium perenne* [13]. The co-cultivation of certain host plant species propagated AMF spores more efficiently than monocultures [11]. Recently, a co-cultivation of maize and sorghum demonstrated suitability as a host consortium in a trap culture [14]. Another study recommended the rotation of host plants in every four cycles of trap culture to maintain original AMF diversities [12]. It is highly likely that the modification of mycotrophic host plant management affects not only the propagation of AMF spores but also the maintenance of original AMF communities in trap cultures.

In agro-ecosystems, AMF communities are strongly influenced by plant diversity. AMF species richness and diversity were significantly higher in a polyculture of crop plants than in a monoculture, whereas AMF community composition was mainly shaped by soil properties [15]. Moreover, forage rotation conserves AMF abundance and AMF taxa in agricultural areas. Differences in AMF community composition were strongly related to changes in crop plant identity [16]. Host plant diversity sustains AMF community compositions by extending AMF-host plant compatibility. Therefore, the exploitation of multiple plant species in trap cultures could improve AMF spore propagation and maintain intact ex situ compositions of AMF communities obtained from natural habitats.

In this trap culture study, we collected rhizo-spheric soil from *Xyris complanata* R.Br., the dominant mycotrophic grass species in a coastal sand dune located in Southern Thailand. The harsh environment in combination with the infertile soil of the sand dune promotes the mutualistic relationship between plants and AMF [1]. Furthermore, AMF diversity in coastal sand dunes is considered relatively high and complex [17,18]. However, in the same habitat, AMF abundance has been reported to vary seasonally, depending on soil moisture content [19,20]. The objectives of this study are to assess seasonal responses of AMF abundance alongside other fungi in starter soil used to prepare trap cultures by general fungal ITS primers and subsequently clarify the effect of different host plant management strategies on the propagation of AMF spores and maintenance of AMF community composition. Our study revealed that AMF spore density and relative abundance were higher in the dry season than in the wet season. Therefore, the coastal sand dune soil was a more suitable starter soil during the dry season. This soil was used under greenhouse conditions in maize and sorghum mono-cultivations and a co-cultivation of maize and sorghum. The co-cultivation of host plants was efficient in terms of AMF spore propagation.

## 2. Materials and Methods

### 2.1. Soil Sample Collection and Nutrient Property Analyses

Soil from *X. complanata* growth was collected from a coastal sand dune located in Ban Tha Kham (7°03′24.4″ N 100°34′56.9″ E) in Hatyai, Songkhla, Thailand. Specimens of dominant plant species found in the study site were collected and sent for identification by a coastal plant taxonomist in the PSU herbarium in the Faculty of Science, Prince of Songkla University, Thailand. Soil samples were randomly collected from three different locations and pooled into a single sample. The soil sample was classified as gleyic arenosols based on the world reference base [21]. Moreover, the soil sample was treated with 10% (*v*/*v*) hydrogen peroxide to remove organic matter for the granulometry analysis. Samples were dry-sieved through 2000-, 1000-, 500-, 180-, 125- and 63-µm meshes. Grain parameters, including mean grain size and sorting, were calculated by the G2SD package v2.1.5 [22] and visualized by the ggplot2 package [23] in R [24].

Six different plots were sampled during both dry and wet seasons in the months of June and November 2021, respectively (*n* = 6 biological replicates). The weather records for the study site were collected from the Thai Meteorological Department (TMD) in 2021. The station representing the study site is WMO Station ID 48569, located in Hatyai (6.55° N, 100.26° E) and +27 m above mean sea level. The weather station was set up to the WMO standard. Monthly temperatures, including maximum, average and minimum temperatures and accumulated rainfall were obtained from TMD.

Soil property analyses were conducted. Soil organic matter was determined by a titration method [25]. Total N was determined by combustion using a C/N analyzer CN628 (LECO, Bangkok, Thailand). Total P and available P were extracted from soil samples using a nitric-perchloric solution in a 1:1 ratio and water, respectively. The amount of P was determined by the molybdovanado-phosphate method using a spectrophotometer (Prove 300, Merck KGaA, Darmstadt, Germany) [26,27]. Total K, magnesium (Mg) and calcium (Ca) were determined by a flame photometric method using inductively coupled plasma-optical emission spectroscopy (ICP-OES) (Avio 500, Perkin Elmer, Waltham, MA, USA) [28]. Sulfur (S) was determined by the turbidity method using a spectrophotometer [29]. Soil pH and electroconductivity were measured with a conductivity meter (Orion Star A112, Thermo Fisher Scientific, Bangkok, Thailand).

### 2.2. Quantification of AMF Spores

AMF spores were extracted from 100 g of soil by wet sieving through sieves with aperture sizes of 500 µm and 38 µm followed by sucrose centrifugation [30]. AMF spores were collected and counted under a bright field microscope. The isolated spores were mounted in polyvinyl lactoglycerol (PVLG) (*n* = 6 biological replicates).

### 2.3. Analysis of Relative Fungal Abundance in Coastal Sand Dunesoil

Total soil DNA from the six different samples (*n* = 6 biological replicates) was isolated using the DNeasy PowerSoil Pro Kit (QIAGEN, Hilden, Germany). The internal transcribed spacer 2 (ITS2) region was amplified using a forward primer containing the sequence 5′-GTG AAT CAT CGA RTC-3′ and a reverse primer containing the sequence 5′-TCC TCC GCT TAT TGA T-3′. The 250 bp paired-end amplicons were sequenced on the Illumina MiSeq platform at GeneWiz (Shanghia, China). Fungal community bioinformatics was performed on the QIIME2 (2021.8 Release) platform [31]. The demultiplexed raw sequence data was trimmed to the ITS2 region with ITSexpress [32] (via q2-itsxpress). Amplicon sequence variants (ASVs) were identified with DADA2 [33], using the Q2-dada2 plugin. A naive-Bayes classifier trained with the UNITE database (version 8.3) [34] was used to assign the fungal taxonomy to the ASVs via a q2-feature-classifier [35]. Sequence variants that were not assigned at the phylum level were filtered out. The data was then imported into Phyloseq [36] in R [24]. The relative abundance of each fungal phylum was visualized with ggplot2 [23]. The data for this study were deposited in the European Nucleotide Archive (ENA) at EMBL-EBI under accession number PRJEB53876.

### 2.4. Investigation of AMF Species in Coastal Sand Dunesoil

A 800 bp small subunit (SSU) region of the ribosomal gene (rDNA) of AMF was amplified by PCR using soil DNA with the mixtures of AML1 (5′-ATC AAC TTT CGA TGG TAG GAT AGA-3′) and AML2 (5′-GAA CCC AAA CAC TTT GGT TTC C-3′) [37]. To amplify the SSU region, PCR was performed in a 20 µL reaction containing 1x HF buffer, 0.2 mM of each dNTPs, 0.5 µM of each of the AML1 and AML2 primers and 1U of Phusion DNA polymerase (Thermofisher Scientific, Waltham, MA, USA). The following cycling conditions were applied: 30 s initial denaturation at 98 °C, 35 cycles of 10 s denaturation at 98 °C, 30 s annealing at 55 °C, 30 s elongation at 72 °C and a 10 min final elongation at 72 °C. PCR products were visualized on 1% agarose gel electrophoresis under UV light.

The PCR products from the six soil DNA samples were equally pooled into a sample that was purified with the QIAquick gel purification kit (Qiagen, Germany). The purified product was ligated with the CloneJet PCR cloning kit (Thermofisher, USA) and transformed into Escherichia coli DH5α. One hundred genuine transformants were selected by colony PCR using the AML1 and AML2 primers. Plasmids were extracted with the BiofactTM Plasmid Mini Prep Kit (Biofact, Daejoen, Korea) and sequenced by Sanger sequencing technology, using the T7 primer.

Sixty-three transformants were submitted for Sanger sequencing. The sequences were annotated by using nucleotide BLAST (blastn) in the NCBI database. Sequences belonging to Glomeromycota were aligned with Clustal Omega [38] and clustered into different operational taxonomic units (OTUs) at a 95% identity threshold using the adegenet [39] and kmer packages [40] in the statistical software R (version 3.6.3). Rarefaction curves were constructed with the vegan package [41] in R to determine whether the number of clones sufficiently represented Glomeromycota diversity at the study site.

The representative AMF OTUs were searched against the NCBI database using BLAST. Ten AMF OTUs from this study, eight AMF reference sequences from the NCBI and MaarjAM databases and two sequences of *Chaetomium globosum* and *Myrothecium* sp. as outgroups were aligned with Clustal Omega [38]. A neighbor-joining tree (Kimura 2 parameters, 1000 replications) was constructed using the MEGA software version 10 [42]. Sequences from this study were made available through the GenBank under accession numbers OP060856–OP060912, OP060817 and OP088733.

### 2.5. Experimental Design for Trap Culture in the Greenhouse

Maize and sorghum were selected as the AMF host plants for the propagation of AMF spores in trap cultures. Eight seedlings of maize, sorghum and a mix of the two species were transplanted into pots (7.5-inch diameter, 6-inch height) containing soil collected from the coastal sand dune mixed with sterile sand and compost in the ratio of 2:1:1 (*v*/*v*). Pots without host plants were set up as controls (*n* = 6 biological replicates). The seedlings and controls were watered daily and fertilized with 200 mL of 0.5× Hoagland solution (3 mM KNO_3_, 0.5 mM NH_4_Cl, 2 mM Ca(NO_3_) × 4H_2_O, 1 mM MgSO_4_, 0.5 mM NH_4_H_2_PO_4_, 46.3 mM H_3_BO_3_, 12.1 mM MnCl_2_, 0.81 mM ZnCl_2_, 0.70 mM CuCl_2_, 0.10 mM Na_2_MoO_4 ×_ 2H_2_O and 0.02% (*w*/*v*) NaFeEDTA) once a week for 10 weeks. After 10 weeks, the pots were air-dried in the greenhouse for 2 weeks to induce AMF sporulation. The trap culture experiment was carried out from July to September 2021 in the greenhouse at temperatures ranging from 24.3 to 36.5 °C and relative humidity ranging from 43.3 to 95.3%. The temperature and relative humidity were recorded by a data logger (HOBO^®^ Pro v2, Bourne, MA, USA). After the completion of the first round of trap culture, a second round of trap culture commenced. Soil from the first trap culture was diluted in a ratio of 1:1 with a new sterile soil and compost mixture. The same host plant species was introduced into the same treatment to propagate the AMF spores. The other half of the soil from the first trap culture and plant roots were collected for determination of AMF spore density and fungal colonization. The second round of trap culture was carried out in the same way as the first round, from November 2021 to January 2022 in the same greenhouse, with recorded temperatures ranging from 22.4 to 35.6 °C and relative humidity from 45.3 to 97.5%.

### 2.6. Measurement of Fungal and AMF Colonization in Roots

Estimation of fungal and AMF root length colonization was performed according to Klinnawee (2020) [20]. 1.5 cm plant roots from each treatment were sampled and cleaned in 1 mL of 10% KOH at 95 °C for 15 min. The roots were rinsed with deionized water and incubated in 1% HCl at room temperature for 10 min. After the removal of the HCl solution, the samples were incubated overnight in 1 mL of Trypan blue staining solution, containing 0.05% (*w*/*v*) Trypan blue, 33% (*v*/*v*) lactic acid and 33% (*v*/*v*) glycerol. The root samples were de-stained twice overnight with 1 mL of 50% (*v*/*v*) glycerol. Thirty root pieces were placed on microscopic slides and mounted in 50% (*v*/*v*) glycerol. One hundred views of roots per cycle were observed under a light microscope (100× magnification) to calculate the percentage of fungal and AMF colonization in both the first and second trap cultures.

### 2.7. Analysis of AMF Communities by T-RFLP

To analyze AMF communities by T-RFLP, DNA samples from the native soil, the second trap culture prepared from different host plant consortiums and the blank were subjected to PCR (*n* = 6 biological replicates). In the first round of PCR, the partial SSU rDNA gene fragment was amplified using AML1 and AML2 primers in the PCR conditions described above [43]. The PCR products obtained were diluted in a ratio of 1:10 and used as templates for the second PCR. In the second PCR, the AML1 and AML2 primers were fluorescently labeled at the 5′ end with HEX and FAM, respectively. A PCR reaction mixture of 100μL was prepared. The purified PCR products were digested separately with the selected restriction enzymes AvaII and HinfI (New England Biolabs, Hertfordshire, UK) for 1 h at 37 °C. Digested products were purified using the QIAquick PCR purification kit (Qiagen, Hilden, Germany) [44].

Terminal restriction fragments (TRFs) from each sample were determined using the 3730/3730xl DNA Analyzer (Applied Biosystem, Waltham, MA, USA) with GeneScan™ 500 LIZ™ dye (Applied Biosystem, Waltham, MA, USA) as the internal size standard. The PeakScanner software v1.0 (Applied Biosystem, Waltham, MA, USA) was used for the analysis of fragment data. Data binning was performed by RawGeno v 2.0 [45] in R [24]. To reduce data noise, only fragments with an intensity above the baseline threshold of 50 fluorescence units were recorded. Relative peak heights were calculated and fragments with an average relative abundance of <5% were excluded [44]. The total number of TRFs represented the species richness in each sample. Jaccard similarity coefficients were calculated for the terminal restriction fragment length polymorphism (T-RFLP) patterns of trap culture soil samples, which were plotted by multidimensional scaling using the ggplot2 package [23] in R [24].

### 2.8. Statistical Analyses

Data was visualized in box plots by the ggplot2 package [23] in the statistical R software [23]. Differences in means of AMF spore density and AMF relative abundance in soils between dry and wet seasons were compared by Student’s *t*-test in R [24]. Significant differences among the means of spore density, fungal colonization, AMF colonization and number of TRFs from the four different treatments of the trap culture experiments were analyzed by one-way ANOVA followed by LSD, using the agricolae package [46] in R [24]. Differences in AMF communities from the four different treatments and the native coastal sand dune soil were analyzed by PERMANOVA using the vegan package [40] in R [24].

## 3. Results

### 3.1. Soil from the Coastal Sand Dunevegetation Was Infertile

According to weather records for 2021, maximum, average and minimum temperatures in Hatyai, Songkhla, Thailand were quite constant over the year (Figure S1A) but total precipitation was highest in November (Figure S1B). Moreover, temperature records over four decades indicate that temperatures in Songkhla Province are relatively higher during April to July [47]. Therefore, in this study, June and November 2021 represented the dry and wet seasons, respectively.

The color of fresh soil from the study site was black due to the organic matter content. However, after hydrogen peroxide treatment, the soil sample was whitish. The microscope showed mainly white and transparent subangular grains that were possibly quartz and felspar (Figure S2A). Granulometric analysis revealed a mean grain size of 541 µm with a sorting of 1.088 (in phi scale). The sample was characterized as poorly sorted coarse sand (Figure S2B).

**Figure 1:**
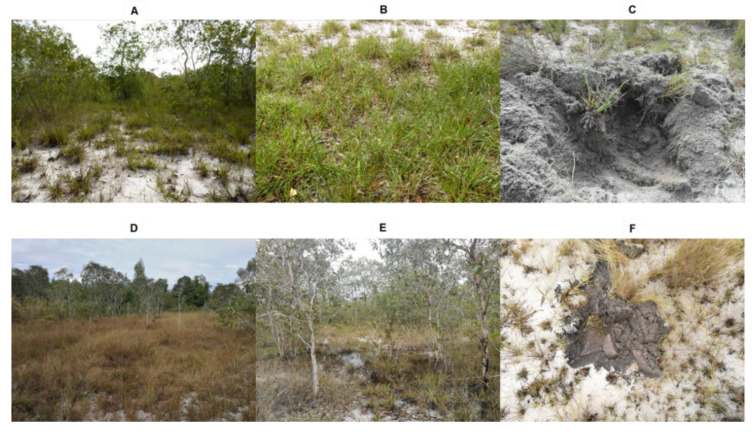
The vegetation of coastal sand dunes during the dry and wet seasons. The coastal sand dune was in Hatyai, Songkhla, Thailand. The vegetation of the study site shows *X. complanata* as the dominant plant species. In the dry season, *X. complanata* and other grasses were green (**A**,**B**). There was no belowground water accumulation (**C**). Conversely, in the wet season, *X. complanata* and other grasses were dry (**D**). Some areas of the study site were flooded (**E**). In non-flooded areas, water accumulated heavily below ground (**F**).

To determine the soil properties of the initial substrate for trap culture, we collected soil around roots of *X. complanata* on the coastal sand dune during both dry and wet seasons. Soil supporting *X. complanata* was chosen because this grass species dominates the coastal sand dune vegetation cover throughout the year (Figure 1). Moreover, the *Xyris* genus was reported to host a high degree of indigenous AMF colonization [48]. The *Xyris* plants from the sample sites were green in the dry season (Figure 1A,B) and withered in the wet season (Figure 1D,E), when some areas of the study site were observed to be waterlogged (Figure 1E). The moisture content of the soil was markedly different between the dry and wet seasons (Figure 1C,F, Table 1). In the wet season, the moisture content of soil collected from non-flooded areas was 20-fold higher than in the dry season (Table 1). In addition to *X. complanata*, *Melaleuca leucadendra* (L.) L., *Melastoma malabathricum* L. and *Baeckea frutescens* L. (C) were also dominant plant species found at the study site (Figure S3).

The coastal sand dune soil contained 0.8% (*w*/*w*) and 1.7% (*w*/*w*) organic matter in the dry and wet season, respectively, which was relatively low compared to organic matter found in other sand dune soils [49,50]. Organic matter and total N both increased during the wet season (Table 1). Seasonal Ca and Mg contents were significantly different. The contents of other soil macronutrients were low and the soil was relatively acidic (Table 1). Due to the soil chemical properties exhibited in the dry and wet seasons, the soil from the study site was considered infertile.

### 3.2. AMF Was More Abundant in the Dry Season

**Figure 2:**
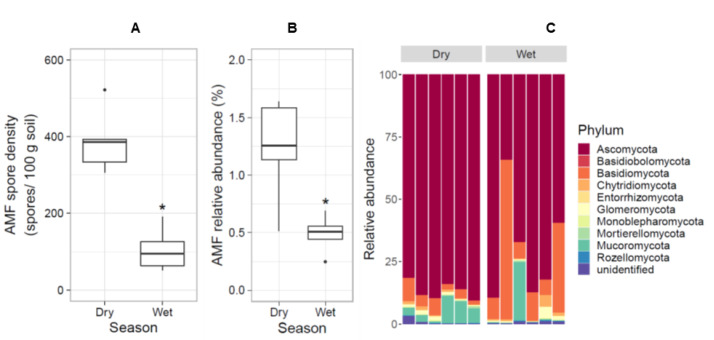
Changes in AMF spore densities and AMF relative abundance between the dry and wet seasons. The box plots show the distribution of AMF spore densities (**A**) and AMF relative abundance (**B**) in soil in the dry and wet seasons (*n* = 6 biological replicates). Fungal relative abundances in soil between dry and wet seasons were determined by analysing ITS2 regions (**C**). Statistical analysis was performed by Student’s *t*-test. Asterisks indicate significant differences (*p* < 0.05).

To determine the seasonal effect on AMF spore density, we counted and compared the number of AMF spores in soil sampled in the dry and wet seasons. The result showed that the number of AMF spores was significantly higher in the dry season (Figure 2A). The abundance of AMF relative to the abundance of other fungi was confirmed by analysis of the fungal ITS2 regions in soil samples. The relative abundance of AMF was low compared to other fungal phyla in the wet season (Figure 2B). The majority of fungi found in the coastal sand dune vegetation belonged to Ascomycota, Basidiomycota and Mucoromycota (Figure 2C). Thus, to maximize the initial number of AMF spores in the starter soil, the trap culture experiment was performed using coastal sand dune soil collected in the dry season.

### 3.3. Glomus Was the Dominant AMF Genus in the Coastal Sand Dune in Dry Season

**Figure 3:**
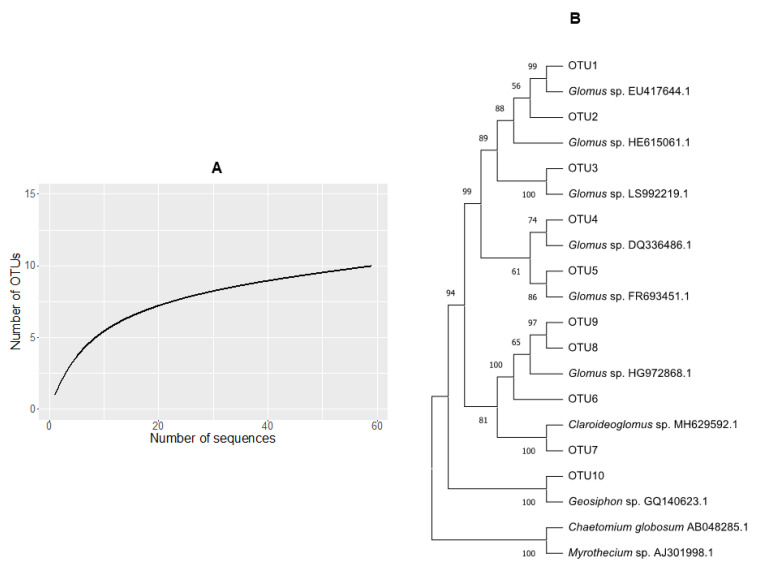
AMF OTUs detected in the soil of coastal sand dune vegetation. The rarefaction curve was plotted from the SSU sequences of Glomeromycota amplified from soil collected from the study sites (**A**). Phytotaxonomic analyses of representative AMF OTUs and their closest sequence similarity from the MaarjAM and NCBI databases were constructed from the SSU sequences of AMF spores present in the soil collected from the study site (1000 bootstrap) (**B**). GenBank accessions are placed behind AMF reference species reported in the databases.

To determine AMF richness in the coastal sand dune vegetation in the dry season, we amplified the AMF specific SSU region of rDNA in the soil samples using AML1 and AML2 primers. After the 63 DNA sequences were annotated, 59 sequences were found to belong to the phylum Glomeromycota. The other four sequences were identified as non-AMF, such as *Mortierella* (KT923281.1) and *Folsomia candida* (KC236239.1). Moreover, to confirm if the numbers of clones sufficiently represented AMF diversity in the sample soils, we constructed rarefaction curves and their extrapolations. The data showed that the rarefaction curves for AMF OTUs reached a plateau (Figure 3A). The result showed that the number of sequences provided full coverage of AMF diversity and a total of 10 OTUs (richness) of the AMF rDNA were detected in the soil samples collected from the study site.

The representative AMF OTU sequences were blasted in the NCBI database and these sequences were used for the phylogenetic analyses. The representative AMF shared a high sequence similarity of 95%. Three Glomeromycota genera were represented: *Glomus*, *Claroideoglomus* and *Geosiphon*. With a relative abundance of 93%, *Glomus* was the most dominant genus, represented by eight OTUs, followed by *Geosiphon* and *Claroideoglomus* represented by only 1 OTU each but with relative abundances of 5% and 2%, respectively (Figure 3B).

### 3.4. Co-Cultivation of Maize and Sorghum Improved the Production of AMF Spores

We hypothesized that the composition of aboveground plant species affects the propagation of AMF spores in a trap culture. Therefore, maize, sorghum and a mixture of maize and sorghum were introduced as host plants in a medium composed of native soil, sterile compost and sand at a ratio of 2:1:1. Pots without plants were prepared as blanks (Figure S4A). All pots, including blanks, were watered daily and fertilized once a week with 200 mL of 0.5x Hoagland’s solution to maintain sufficient nutrients for the growth of the host plants. The seedlings were maintained for 10 weeks (Figure S4B). Later, watering was omitted for the induction of AMF sporulation. Moreover, to determine the compatibility of host plants and indigenous endophytic fungi including AMF in the trap culture, fungal and AMF colonizations were determined in the roots of seedlings after 10 weeks of cultivation.

**Figure 4:**
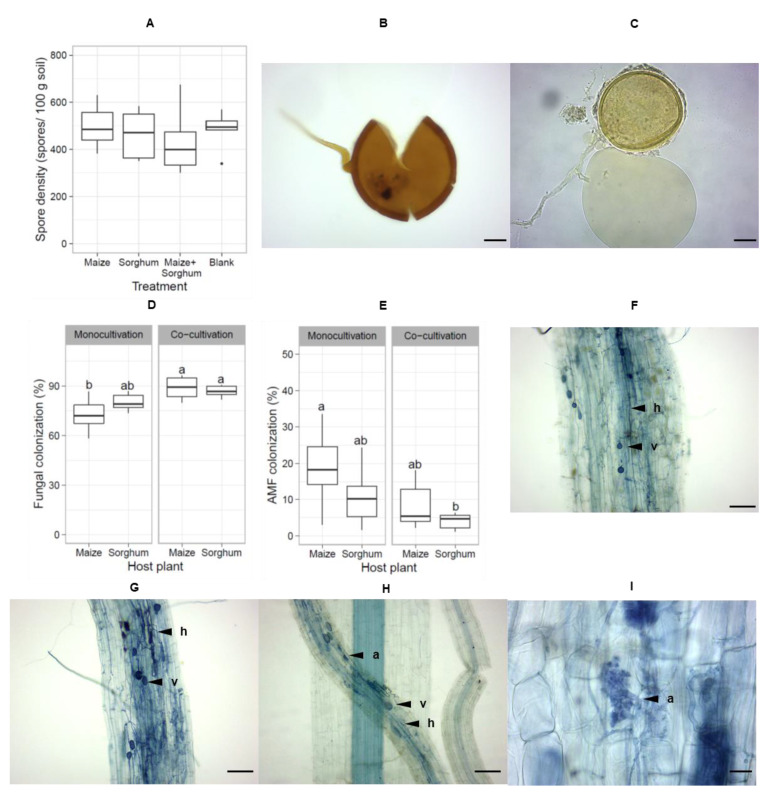
AMF spore density and root length colonization by fungi and AMF after the first trap culture. AMF spore densities were counted from mono-cultivation trap cultures containing maize or sorghum, a co-cultivation trap culture containing maize and sorghum and a trap culture without host plants as a blank (**A**). *Glomus* spores were observed under the light microscope (40× objective magnification, scale = 20 µm) (**B**,**C**). Degrees of fungal colonization (**D**) and AMF colonization (**E**) in roots from each treatment were compared. Mycorrhization structures such as hyphae (**h**), vesicle (**v**) and arbuscule (**a**) were observed under the light microscope (150 views, 10× objective magnification, scale = 100 µm) (**F**–**H**). Mature arbuscules were present in the host roots (scale = 20 µm) (**I**). The box plots show the distribution of data in each trap culture condition (*n* = 6 biological replicates). Statistical analysis was performed by one-way ANOVA following LSD. Different letters indicate significant differences (*p* < 0.05).

AMF spores were extracted from the soil in each treatment and counted. Differences in AMF densities among treatments were not detected after the first trap culture. AMF spore density was not significantly higher in the mono- and co-cultivations than in the blank treatment (Figure 4A). All the AMF spores observed under the microscope were *Glomus* spores (Figure 4B,C). Furthermore, there was no significant difference in fungal colonization between maize and sorghum roots, but fungal colonization of maize and sorghum roots was higher in the co-cultivation than in the mono-cultivation (Figure 4D). However, AMF colonization of roots between plant species and trap culture systems was not different. AMF colonized 5–35% of roots (Figure 4E). Various mycorrhization structures were observed along the roots, including hyphae, vesicles and arbuscules (Figure 4F–H). Mature arbuscules were also detected (Figure 4I). Therefore, these results suggest that the propagation of AMF spores was not successful in the first trap culture over 12 weeks in the greenhouse. However, the presence of AMF structures in the host plant roots within 10 weeks demonstrated that maize and sorghum could be colonized by AMF in the trap culture system.

**Figure 5:**
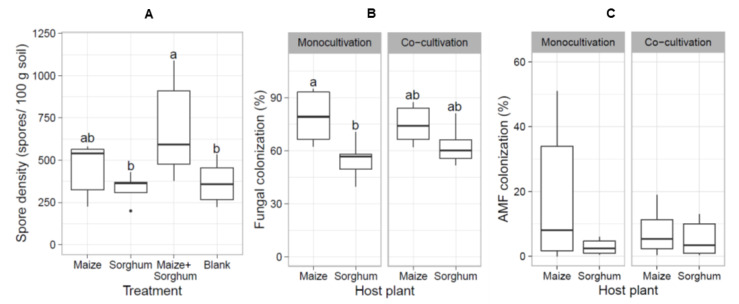
AMF spore density and root length colonization by fungi and AMF after the second trap culture. The box plots show the distribution of AMF spore density in mono-cultivation trap cultures containing maize or sorghum; a co-cultivation trap culture containing maize and sorghum; and a trap culture without host plants as a blank (**A**); fungal colonization (**B**); and AMF colonization (**C**) in roots from each treatment (*n* = 6 biological replicates). Statistical analysis was performed by one-way ANOVA following LSD. Different letters indicate significant differences (*p* < 0.05).

To increase spore density in the trap culture, a second trap culture was prepared using the same host plants with the same treatments as the first trap culture. At the end of the second trap culture, spore density and root length colonization by fungi and AMF were determined. The results showed that the co-cultivation of maize and sorghum significantly increased spore density compared with the blank treatment, while mono-cultivation of maize and sorghum did not (Figure 5A). The highest spore density in the co-cultivation trap culture indicated that co-cultivation of maize and sorghum promoted the propagation of AMF spores. A significant difference between fungal colonization of maize and sorghum was detected in the mono-cultivation but not in the co-cultivation (Figure 5B). However, AMF colonization of both host plant roots showed no significant difference in both systems (Figure 5C). These results demonstrated that an increase in spore density in the trap cultures was not consistent with the degree of AMF colonization.

### 3.5. Trap Culture in the Greenhouse Shifted AMF Communities of Coastal Sand Dune Soil

**Figure 6:**
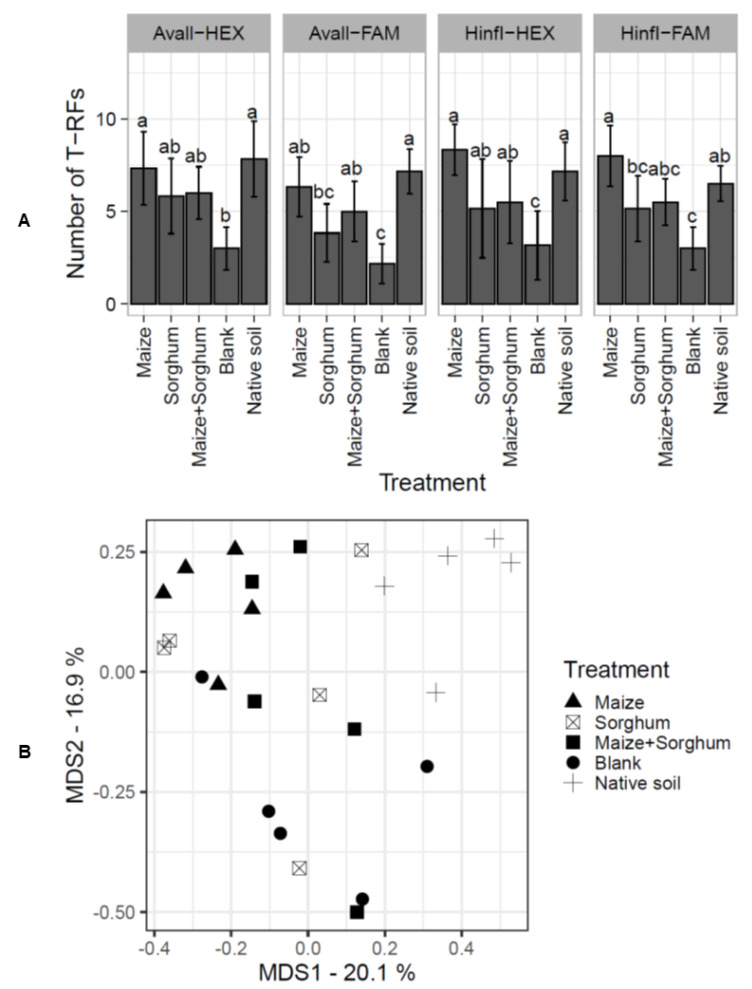
AMF community in trap cultures. 800-bp SSU regions of AMF from various trap cultures, including maize, sorghum, maize + sorghum, blank and native soil, were amplified by the HEX-labelled AML1 primer and FAM-labelled AML2 primer. The terminal restriction fragments (TRFs) in each sample (*n* = 6 biological replicates) were derived from the digestion of PCR products by AvaII and HinfI (**A**). Statistical analysis was performed by one-way ANOVA following LSD. Different letters indicate significant differences (*p* < 0.05). The multidimensional scaling plot of AMF community compositions (*n* = 5–6 biological replicates) found in the trap culture soils was generated based on the presence and absence of TRFs from the 5ʹ-HEX-labelled PCR products digested with AvaII and HinfI (**B**).

To compare the AMF soil communities between different trap cultures and the native coastal sand dune soil, we analysed AMF communities by T-RFLP. We used the HEX-labelled AML1 primer and the FAM-labelled AML2 primer in the amplification of SSU fragments of AMF and AvaII and HinfI as the digestion enzymes. The total number of different TRFs was used to compare AMF community diversity. The result showed that patterns of TRFs generated from the different fluorescent markers and digestion enzymes tended to be consistent. The number of TRFs was lowest in the blank (Figure 6A). Seventy TRFs were generated by the AML1 primer labelled with HEX at the 5′ end, while the AML2 primer labelled with FAM at the 3′ end generated 68 TRFs. Therefore, the AML1 fragments were used for multidimensional scaling plots of AMF community compositions. AMF community compositions in trap cultures maintained in the greenhouse conditions were significantly different from those in the native coastal sand dune soil (Figure 6B and Table 2). Therefore, co-cultivation of maize and sorghum in the trap culture did not minimize changes in the native AMF communities.

## 4. Discussion

### 4.1. Soil in the Coastal Sand Dune Was Infertile

The soil from the coastal sand dune used in the trap culture was composed of 3 ppm of available N derived from NO_3_^−^ and NH_4_^+^, 25 ppm of available P and 16 ppm total K. These soil nutrient levels are markedly lower than those that would be found in agricultural soils. For example, soil in a citrus orchard used for the preparation of a trap culture contained 284 ppm of available N, 69 ppm of available P and 65 ppm of available K [11]. Furthermore, in our previous studies, we collected soil from organic lowland rice paddies for trap cultures. Paddy soil is generally considered a low P soil, but the collected soil contained 76 ppm total P, which is approximately three-fold the total P of the coastal sand dune soil used in the present study [20,51]. The macronutrient contents of the soil in the present study are also lower than those reported for coastal forest soils [49]. In coastal soil, P is particularly scarce and gradually decreases along the depth of the soil [52]. Therefore, nutrient deficiencies, especially of P, could be important limiting factors for plants in coastal ecosystems.

Some areas of the study site were waterlogged in the wet season (Figure 1E). Soil moisture was significantly higher than in the dry season. The increase in gravimetric water content was consistent with the higher accumulation of soil organic matter and soil N (Table 1). Changes in organic matter and total N in coastal sand dune soil located in Thailand are connected with the dynamics of soil C, soil N and organic soil P in lowland tropical forest soils [53]. In addition, precipitation is positively correlated with soil organic carbon and with soil microbial biomass [54]. On tropical coastal sand dunes, the higher precipitation in the wet season affects soil properties, especially organic matter content and total N. Nevertheless, the soil is extremely nutrient poor in both the dry and wet seasons.

### 4.2. AMF Is Abundant in the Coastal Sand Dune Soil in the Dry Season

AMF spores and relative abundance in the coastal sand dune soil were low in the wet season (Figure 2A,B), when soil moisture is an important factor that limits AMF colonization and shapes fungal and AMF communities in plant root systems [20]. Reduced AMF colonization due to flooding impairs AMF symbiosis but not AMF viability [55]. AMF spore density, however, was higher in sugarcane fields in the summer than in the monsoon season, which has the highest precipitation [56] and higher AMF spore densities accumulate in upland rice paddy soils than in lowland rice paddy soils. Moreover, among the AMF spores detected in a study of coastal wetland soil, the proportion of live to dead AMF spores was significantly lower in the wet season [57]. In our study, we found that organic matter, total N and total P increased in the wet season (Table 1) and it may be the difference in the availability of these nutrients in the coastal sand dune soil that is responsible for the seasonal difference in AMF sporulation [56].

Based on our ITS sequence analysis, Ascomycota and Basidiomycota were the two most abundant fungi in the coastal sand soil in both dry and wet seasons. However, the relative abundance of Basidiomycetes was higher in the wet season (Figure 2C). ITS sequences of Ascomycota, Basidiomycota and Glomeromycota have been detected in another study of coastal sand dune vegetation [58], where the most common and abundant fungi in the vegetation were Ascomycota and Basidiomycota. Glomeromycota were identified in only two to six percent of the fungi. Moreover, the difference between soil moisture content in upland and lowland rice paddies alters the relative abundance of Ascomycota and Basidiomycota and was reported to significantly reduce not only the relative abundance of Glomeromycota but also its species richness and diversity [59]. The minority of AMF compared with other fungi in soil compositions, especially in high-moisture soils, indicates that the selection of starter soil for the preparation of a trap culture is vital for the successful propagation of the AMF inoculum, especially when the aim is to produce a synthetic AMF community.

### 4.3. Plant Communities Affect AMF Communities in Coastal Sand Dune Ecosystems

The coastal sand dune soil in this study was mainly covered by *X. complanata*. There were 10 OTUs representing three AMF genera in the soil. *Glomus* was identified as the dominant genus in this habitat (Figure 3A,B). In a study conducted in Portugal, coastal sand dune soil covered by monospecific stands of *Ammophila arenaria* also harbored only three AMF genera and *Glomus* was the most abundant of them [60]. However, in the southern region of Brazil, coastal sand dune soil with low anthropogenic disturbance, covered with poly-specific stands of grass species, harbored 25 AMF species were detected from 7 AMF genera [18]. In the north-eastern region of Brazil, 34 AMF species from 14 AMF genera were detected in sand dunes with high anthropogenic pressure [17]. *Gigaspora* fungi were the most abundant AMF in both these habitats.

### 4.4. Co-Cultivation of Host Plants Improves the Production of AMF Spores in Trap Culture

The first trap culture failed to increase AMF spore density on maize and sorghum roots (Figure 4A), although the host plants were colonized fully by fungi and moderately by AMF (Figure 4D,E). Two successive cycles of trap culture are necessary for the production of AMF spores under greenhouse conditions. Many studies have found that few AMF spores can be detected after one trap culture [61,62,63]. Therefore, one trap culture is not sufficient to propagate AMF spores ex-situ in soil under greenhouse conditions.

In the second trap culture, co-cultivation of the host plants produced more AMF spores than mono-cultivations of the host plants (Figure 5A). This indicated that the aboveground plants affected AMF sporulation. In our trap cultures, maize was a more efficient AMF host than sorghum because AMF spore density at the end of the second trap culture had not increased in the sorghum mono-cultivation (Figure 5A). Yao et al. (2010) reported that co-cultivation of maize, sorghum and clover was the most compatible combination to produce AMF spores in trap culture [11]. AMF spore production was not better in a co-cultivation of maize and sorghum than in mono-cultivations of maize, sorghum, or clover. Moreover, a co-cultivation of sorghum and clover yielded the fewest AMF spores. The correct selection of host plants in a trap culture leads to the successful propagation of AMF spores since the outcome depends on plant species-by-environment-by-AMF-by-management interactions. Additionally, the co-cultivation of maize and sorghum generated interspecific competition between the host plants. This might have induced AMF sporulation in the pots since the density of AMF spores below ground is consistent with the intensity of interspecific competition among invasive and native plants above ground [64].

In our experiment, the second trap culture was completed 140 days after the planting of host plants in the coastal sand dune soil. While only 10% of the host plant roots were occupied by AMF, 60–90% were colonized by fungi (Figure 5B,C). In a previous work, inoculation of sorghum plants in a 120-day trap culture for AMF propagule production enabled AMF to colonize up to 6.5% of the host roots [62]. In an earlier trap culture study [11], maize, sorghum and clover were grown in a greenhouse in 500 g of medium containing agricultural soil and sterile sand in a ratio of 1:1. After 120 days, there was no difference in AMF root colonization among mono-, bi- and tri-cultivation treatments. Furthermore, under field conditions, no differences in AMF colonization were detected in summer squash and eggplant grown in monocultures and polycultures [15]. Therefore, the co-cultivation of AMF host plants increases spore production but not the degree of AMF colonization in their roots.

Mono- and co-cultivation of host plants sustained the native AMF community to a certain extent.

The number of TRFs in soil is an indicator of AMF species richness [44]. The lowest number of TRFs was detected in the blank treatment. Since AMF are obligate symbionts, the blank treatment was set as a negative control in this experiment (Figure 6A). This result indicates that, within 120 days, the absence of a host plant significantly reduced AMF species richness in the soil of the first and second trap cultures. It was reported that indigenous AMF species richness and abundance in soil from a tall prairie grass habitat were substantially reduced under greenhouse conditions using sorghum as the host plant [65]. However, almost all native host plants are capable of producing higher amounts of AMF spores than sorghum [65]. Furthermore, invasion of non-native plants into natural habitats reduces AMF abundance and species richness [66]. This suggests that AMF-associated host plants play an important role in maintaining AMF species composition in trap cultures. Hence, the absence of host plants in the blank treatment markedly affected the AMF species richness.

The AMF communities in trap cultures differed significantly from the AMF communities in native soil. Co-cultivation of maize and sorghum did not attenuate the change in the AMF community (Figure 6B and Table 2). Loss of AMF diversity and maintenance of AMF composition in trap cultures under greenhouse conditions remain intractable problems in trap culture soils [67]. On the other hand, trap culture causes the alteration of AMF composition in the starter soil by introducing some AMF species or recovering some hidden AMF species [68]. Furthermore, trap culture of AMF propagules from natural habitats brings about the detection of some AMF species described as cryptic since they are not initially detected in the starter soil [14,69].

AMF composition in trap culture is influenced by intricate plant species-by-environment-by-AMF-by-management interactions. In growth conditions and soil properties that were completely different from those of the coastal sand dune ecosystem, maize and sorghum host plants were unable to maintain the AMF community associated with the native plant host species in coastal sand dune soil. However, co-cultivation of the host plants was an effective approach to enhance AMF spore production in the trap culture.

## 5. Conclusions

The sampling site on a coastal sand dune in Songkhla, Thailand was a nutrient-poor habitat, dominantly covered by the grass species *X. complanata*. Almost all the AMF detected belonged to the genus *Glomus*. The soil chemical properties, especially organic matter, total N and moisture content, fluctuate seasonally. The low soil fertility and gravimetric content enhanced AMF spore density and relative abundance in the soil. Thus, the soil in the dry season was more suitable as the AMF inoculant for propagation of AMF spores. In trap cultures under greenhouse conditions, a co-cultivation of maize and sorghum provided more efficient propagation of AMF spores than mono-cultivations of maize or sorghum. However, none of the trap cultures could entirely maintain the AMF community composition present in the coastal sand dune soil. Therefore, our result suggests that a consortium of AMF host plants could increase the efficiency of AMF propagation by trap culture, which is an important step in the production of AMF biofertilizers.

## Figures and Tables

**Table 1:** Chemical properties of soil collected from the study site in the dry and wet seasons.

Parameter (Unit)	Dry Season	Wet Season
Organic matter (%*w*/*w*)	0.8 ± 0.3	1.7 ± 0.4 *
Total N (ppm)	418.5 ± 227.0	837.7 ± 271.3 *
NO_3_^−^ (ppm)	8.6 ± 3.9	10.7 ± 5.1
NH_4_^+^ (ppm)	2.9 ± 2.1	2.8 ± 0.8
Total P (ppm)	16.8 ± 2.8	25.8 ± 17.2
Available P (ppm)	13.0 ± 2.9	16.6 ± 12.3
K (ppm)	21.6 ± 16.4	9.0 ± 2.3
Ca (ppm)	57.9 ± 9.4	43.1 ± 8.0 *
Mg (ppm)	8.9 ± 1.7	5.2 ± 0.4 *
S (ppm)	20.9 ± 8.2	20.5 ± 17.1
pH	5.2 ± 0.3	5.5 ± 0.3
EC (dS/m)	0.010 ± 0.002	0.011 ± 0.001
Moisture content (%*w*/*w*)	0.7 ± 0.2	19.6 ± 1.2 *

The data represent the mean and standard deviation (*n* = 6 biological replicates). Statistical analysis was performed by Student’s *t*-test. Asterisks indicate significant differences between the dry and wet seasons (*p* < 0.05).

**Table 2:** Comparison of T-RF components in trap culture under different host plants. Comparison was performed by pairwise PERMANOVA of Jaccard distances.

Comparison	R^2^	*p*-Value
Native soil × Maize	0.36702	0.0083 **
Native soil × Sorghum	0.29207	0.0171 *
Native soil × Maize + Sorghum	0.22444	0.017 *
Native soil × Blank	0.3121	0.0089 **
Maize × Sorghum	0.12514	0.3605
Maize × Maize + Sorghum	0.14634	0.1581
Maize × Blank	0.27034	0.0092 **
Sorghum × Maize + Sorghum	0.09452	0.6704
Sorghum × Blank	0.17119	0.0775
Maize + Sorghum × Blank	0.15955	0.0677

Significant codes: * *p* < 0.05, ** *p* < 0.01.

## Data Availability

Not applicable.

## Supplementary Materials

The following supporting information can be downloaded at: https://www.mdpi.com/article/10.3390/jof8101061/s1, Figure S1. Monthly temperature and precipitation in 2021 in Hatyai, Songkhla, Thailand, Figure S2. Characteristics of the coastal sand dune soil, Figure S3. Examples of dominant plant species found in the coastal sand dune vegetation, Figure S4. Trap culture for AMF spore propagation in the greenhouse.
