# Supplementary material for: Dry-Season Soil and Co-Cultivated Host Plants Enhanced Propagation of Arbuscular Mycorrhizal Fungal Spores from Sand Dune Vegetation in Trap Culture
Source: J Fungi (Basel). 2022 Oct 10;8(10):1061. doi: 10.3390/jof8101061 (PMC9604700; doi:10.3390/jof8101061)
Supplement: Supplementary file 1 [file jof-08-01061-s001.zip › jof-1902322-supplementary.pdf]

Journal of *Fungi*

MDPI

Supplementary

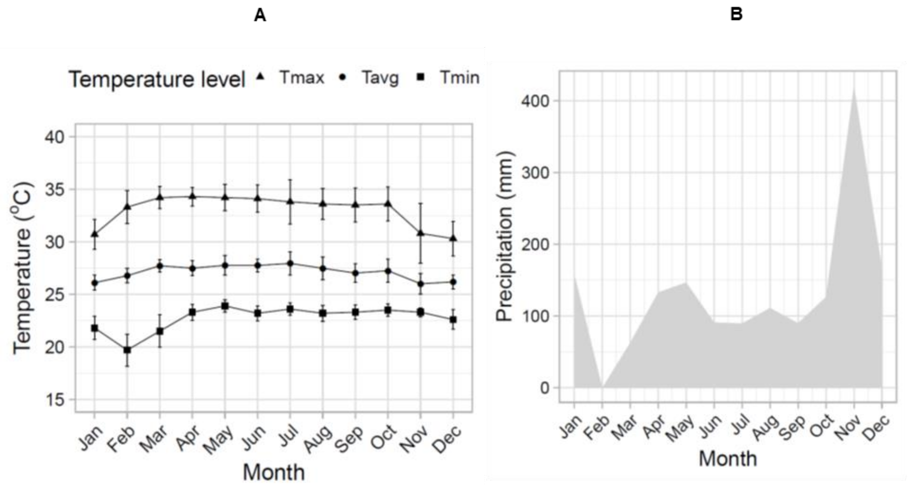

Figure S1. Monthly temperature and precipitation in 2021 in Hatyai, Songkhla, Thailand. In this study, June and November were considered as the dry and wet seasons. Each month's average maximum temperature (Tmax), average temperature (Tavg), minimum temperature (Tmin) and total precipitation (B) were displayed. The error bars represent the standard deviation.

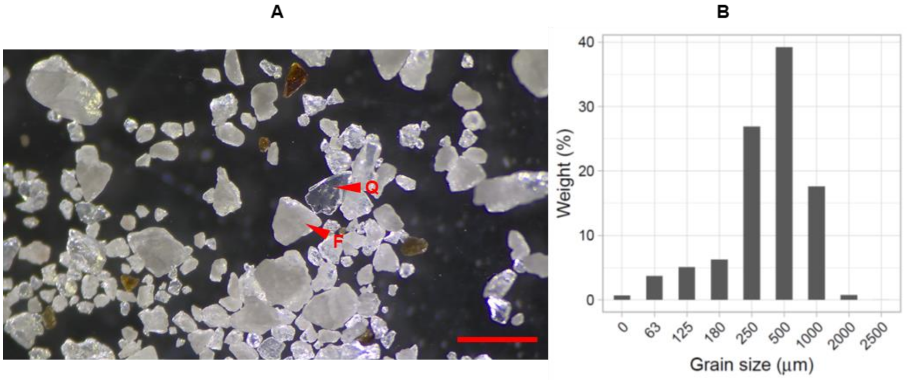

Figure S2. Characteristics of the coastal sand dune soil. The soil sample was treated with hydrogen peroxide and visualized under a stereomicroscope. Quartz (Q) and felspar (F) were detected in the soil (scale = 1 mm) (A). The granulometric analysis shows the distribution of grain size in the coastal sand soil at the study site (B).

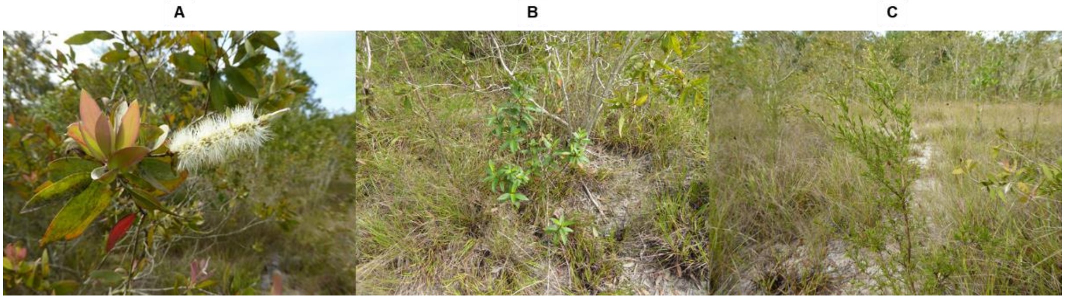

Figure S3. Examples of dominant plant species found in the coastal sand dune vegetation. Apart from *X. complanata*, **the dominant plant species in the study site were** *Melaleuca leucadendra* (L.) L. (A), *Melastoma malabathricum* L. (B), *Baeckea frutescens* L. (C).

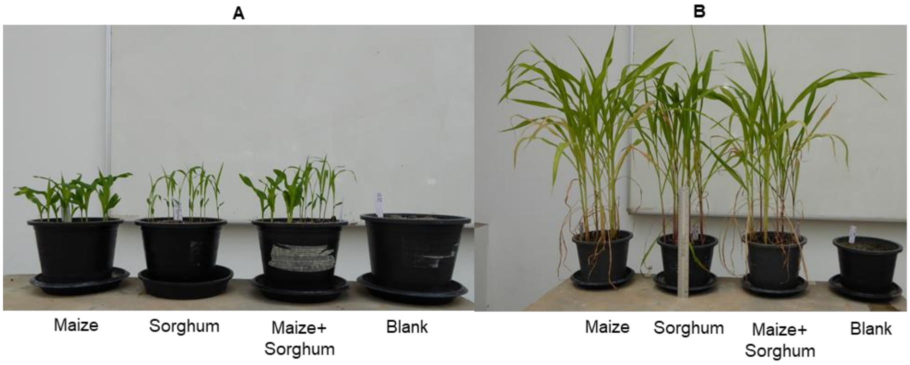

Figure S4. Trap culture for AMF spore propagation in the greenhouse. Coastal sand soil was mixed with sterile sand and compost in a ratio of 2:1:1. Maize, sorghum and a mixture of maize and sorghum were grown as host plants. Pots without seedlings were used as a negative control (A). The seedlings were grown in an open greenhouse for 10 weeks (B). Subsequently, the trap cultures were air-dried to induce AMF sporulation.
